# 4-Iodo-3,3′-dimethoxy­biphen­yl

**DOI:** 10.1107/S1600536808019557

**Published:** 2008-07-05

**Authors:** Qamar Ali, Zahid Hussain, Muhammad Raza Shah, Donald VanDerveer

**Affiliations:** a HEJ Research Institute of Chemistry, International Center for Chemical & Biological Sciences, University of Karachi, Karachi 75270, Pakistan; b Chemistry Department, Clemson University, Clemson, SC 29634-0973, USA

## Abstract

Mol­ecules of the title compound, C_14_H_13_IO_2_, exhibit no π–π inter­actions. The dihedral angle between the two aromatic rings is 43.72 (9)°. The shortest inter­molecular I⋯O distance is 3.408 (2) Å, which is significantly less than the sum of the van der Waals radii for I and O (3.50 Å).

## Related literature

For related literature, see: Litvinchuk *et al.* (2004 ▶); Baudry *et al.* (2006 ▶); Sisson *et al.* (2006 ▶); Ali *et al.* (2008 ▶); Ibad *et al.* (2008 ▶); Baumeister *et al.* (2001 ▶).
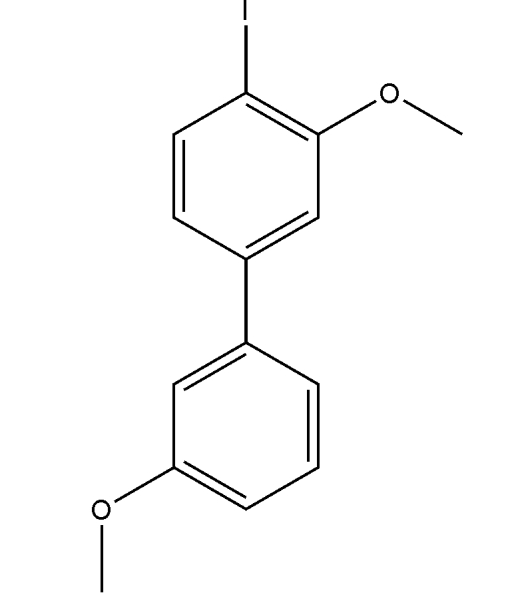


## Experimental

### 

#### Crystal data

C_14_H_13_IO_2_
*M* *_r_* = 340.14
Monoclinic, 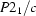

*a* = 11.932 (2) Å
*b* = 15.382 (3) Å
*c* = 6.9940 (14) Å
β = 90.68 (3)°
*V* = 1283.6 (4) Å^3^
*Z* = 4
Mo *K*α radiation
μ = 2.48 mm^−1^
*T* = 153 (2) K
0.43 × 0.38 × 0.36 mm

#### Data collection

Rigaku Mercury CCD diffractometer
Absorption correction: multi-scan (Jacobson, 1998 ▶) *T*_min_ = 0.415, *T*_max_ = 0.469 (expected range = 0.362–0.409)
9179 measured reflections
2337 independent reflections
2206 reflections with *I* > 2σ(*I*)
*R*_int_ = 0.021

#### Refinement

*R*[*F* ^2^ > 2σ(*F* ^2^)] = 0.022
*wR*(*F* ^2^) = 0.053
*S* = 1.09
2337 reflections
156 parameters
H-atom parameters constrained
Δρ_max_ = 1.19 e Å^−3^
Δρ_min_ = −0.45 e Å^−3^

### 

Data collection: *CrystalClear* (Molecular Structure Corporation & Rigaku, 2006 ▶); cell refinement: *CrystalClear*; data reduction: *CrystalClear*; program(s) used to solve structure: *SHELXTL* (Sheldrick, 2008 ▶); program(s) used to refine structure: *SHELXTL*; molecular graphics: *SHELXTL*; software used to prepare material for publication: *SHELXTL*.

## Supplementary Material

Crystal structure: contains datablocks I, global. DOI: 10.1107/S1600536808019557/bt2736sup1.cif

Structure factors: contains datablocks I. DOI: 10.1107/S1600536808019557/bt2736Isup2.hkl

Additional supplementary materials:  crystallographic information; 3D view; checkCIF report

## Figures and Tables

**Fig. 1.**
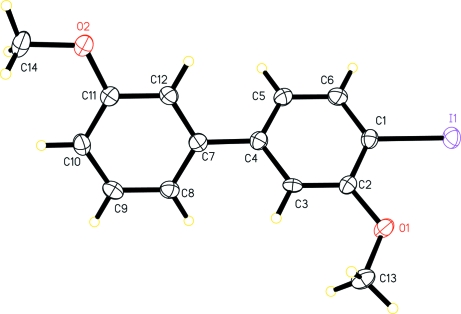
A perspective thermal elipsoid drawing (50% probability) of the molecule showing the atom labelling scheme.

**Fig. 2.**
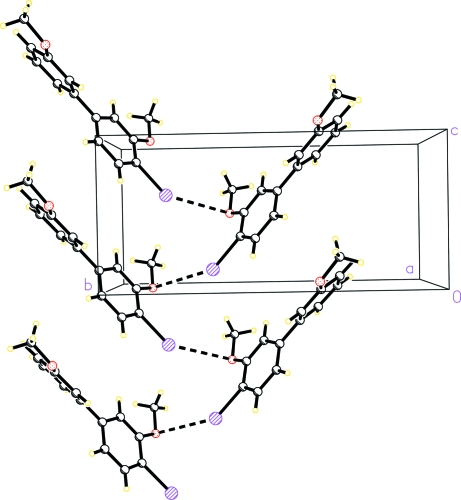
A packing diagram along the a axis showing the short I - O contacts with dashed lines.

## supplementary crystallographic information

### Comment

Self-assembling molecules based on oligo(*p*-phenylene)s are receiving increased attention as building blocks for supramolecular structures, such as artficial ion channels (Litvinchuk *et al.*, 2004; Baudry *et al.*,2 006). One can envision that incorporation of a conjugated macrocycle such as porphyrin into an oligo(*p*-phenylene)s, extends the cylindrical supramolecular organization capabilities of the oligo(*p*phenylene)s (Sisson *et al.*, 2006) and can result in functionalized pores. The titled compound can be used as a precursor for the synthesis of oligo(*p*-phenylene)s (Baumeister  *et al.*, 2001).  The I1—O1 intermolecular distance  is 3.408 (2) Å which is significantly less than 3.50 Å, the sum of the van der Waals radii for I and O. Reported data (Ali *et al.*, 2008)  indicate  that the oxygen atom of a methoxy group polarizes the electronic cloud surrounding the iodide causing a reduction in the I—O intermolecular distance. The phenyl rings are   twisted by a dihedral angle of 43.72 (9)°, which is typical for biphenyl molecules (Ibad *et al.*, 2008). The presence of a  iodo group at only one phenyl ring least to a twist between the two rings, whereas the rings are coplanar when  both phenyl rings bore a iodo group (Ali *et al.*, 2008). The crystal packing diagram (Fig.2) shows that the molecules are interlinked by I—O interactions.

### Experimental

5 g (10.7 mmol) of 4,4`-diiodo-3`-methoxy[1,1`-biphenyl]-3-yl-methyl ether was dissolved in 30 ml of THF in a 250 ml round bottom flask. The reaction mixture was stirred until a clear solution formed. Then a *tert*-butyl lithium solution (8.2 ml, 1.7 *M* in pentane 13.9 mmol) was added at 0 C. The reaction was monitored after an interval of 5 minutes through TLC.The reaction was stirred for thirty five minutes until a spot of 4-iodo-3,3`-dimethoxy-1,1`-biphenyl appeared on the TLC and was then quenched with 10 ml (1 N HCl) and extracted with 30 ml of chloroform three times. The crude reaction mixture was concentrated using a rotary evaporator. A super saturated solution of crude reaction mixture was prepared in chlorofrom and then methanol was added to this super-saturated solution of reaction mixture, two layers were formed which were separated and analysed by TLC (Hexane:Chloroform 1:1). The methanol layer contained 4-iodo-3,3'-dimethoxybiphenyl as the major product. The slow evaporation of methanol at room temperature yielded colorless crystals.

### Refinement

All H atoms were geometrically positioned and allowed to ride on the corresponding parent atom with C—H = 0.96 Å and *U*_iso_(H) = 1.5*U*_eq_(C_methyl_) or 1.2*U*_eq_(C_aromatic_), respectively. The methyl groups were allowed to rotate but not to tip.

### Crystal data

**Table:**

C_14_H_13_IO_2_	*F*_000_ = 664
*M**_r_* = 340.14	*D*_x_ = 1.760 Mg m^−^^3^
Monoclinic, *P*2_1_/*c*	Mo *K*α radiation λ = 0.71073 Å
*a* = 11.932 (2) Å	Cell parameters from 4368 reflections
*b* = 15.382 (3) Å	θ = 2.9–26.4º
*c* = 6.9940 (14) Å	µ = 2.48 mm^−^^1^
β = 90.68 (3)º	*T* = 153 (2) K
*V* = 1283.6 (4) Å^3^	Chip, colorless
*Z* = 4	0.43 × 0.38 × 0.36 mm

### Data collection

**Table:**

Rigaku Mercury CCD diffractometer	2337 independent reflections
Radiation source: Sealed Tube	2206 reflections with *I* > 2σ(*I*)
Monochromator: Graphite Monochromator	*R*_int_ = 0.021
Detector resolution: 14.6306 pixels mm^-1^	θ_max_ = 25.4º
*T* = 153(2) K	θ_min_ = 3.2º
ω scans	*h* = −14→14
Absorption correction: multi-scan(Jacobson, 1998)	*k* = −18→18
*T*_min_ = 0.415, *T*_max_ = 0.469	*l* = −8→6
9179 measured reflections	

### Refinement

**Table:**

Refinement on *F*^2^	Secondary atom site location: difference Fourier map
Least-squares matrix: full	Hydrogen site location: inferred from neighbouring sites
*R*[*F*^2^ > 2σ(*F*^2^)] = 0.022	H-atom parameters constrained
*wR*(*F*^2^) = 0.054	*w* = 1/[σ^2^(*F*_o_^2^) + (0.0234*P*)^2^ + 1.6489*P*] where *P* = (*F*_o_^2^ + 2*F*_c_^2^)/3
*S* = 1.10	(Δ/σ)_max_ = 0.001
2337 reflections	Δρ_max_ = 1.19 e Å^−^^3^
156 parameters	Δρ_min_ = −0.45 e Å^−^^3^
Primary atom site location: structure-invariant direct methods	Extinction correction: none

### Special details

**Table:**

Geometry. All e.s.d.'s (except the e.s.d. in the dihedral angle between two l.s. planes) are estimated using the full covariance matrix. The cell e.s.d.'s are taken into account individually in the estimation of e.s.d.'s in distances, angles and torsion angles; correlations between e.s.d.'s in cell parameters are only used when they are defined by crystal symmetry. An approximate (isotropic) treatment of cell e.s.d.'s is used for estimating e.s.d.'s involving l.s. planes.
Refinement. Refinement of *F*^2^ against ALL reflections. The weighted *R*-factor *wR* and goodness of fit *S* are based on *F*^2^, conventional *R*-factors *R* are based on *F*, with *F* set to zero for negative *F*^2^. The threshold expression of *F*^2^ > σ(*F*^2^) is used only for calculating *R*-factors(gt) *etc*. and is not relevant to the choice of reflections for refinement. *R*-factors based on *F*^2^ are statistically about twice as large as those based on *F*, and *R*- factors based on ALL data will be even larger.

### Fractional atomic coordinates and isotropic or equivalent isotropic displacement parameters (Å^2^)

**Table:**

	*x*	*y*	*z*	*U*_iso_*/*U*_eq_	
I1	0.351251 (14)	0.680339 (10)	0.13178 (2)	0.02636 (8)	
C1	0.3045 (2)	0.59482 (15)	0.3506 (3)	0.0207 (5)	
C2	0.37606 (19)	0.58357 (15)	0.5087 (3)	0.0187 (5)	
C3	0.34448 (17)	0.52906 (15)	0.6519 (3)	0.0158 (4)	
H3A	0.3926	0.5222	0.7618	0.019*	
C4	0.2424 (2)	0.48233 (15)	0.6425 (3)	0.0203 (5)	
C5	0.1732 (2)	0.49371 (17)	0.4818 (4)	0.0251 (5)	
H5A	0.1037	0.4625	0.4718	0.030*	
C6	0.2044 (2)	0.55002 (17)	0.3361 (4)	0.0250 (5)	
H6A	0.1566	0.5577	0.2261	0.030*	
C7	0.2107 (2)	0.42139 (15)	0.7984 (3)	0.0203 (5)	
C8	0.2903 (2)	0.36430 (16)	0.8774 (3)	0.0219 (5)	
H8A	0.3663	0.3652	0.8341	0.026*	
C9	0.2583 (2)	0.30671 (16)	1.0183 (4)	0.0233 (5)	
H9A	0.3129	0.2672	1.0707	0.028*	
C10	0.1492 (2)	0.30429 (16)	1.0864 (4)	0.0235 (5)	
H10A	0.1285	0.2638	1.1844	0.028*	
C11	0.0710 (2)	0.36164 (16)	1.0095 (4)	0.0226 (5)	
C12	0.1018 (2)	0.41935 (16)	0.8648 (4)	0.0222 (5)	
H12A	0.0468	0.4580	0.8107	0.027*	
C13	0.5475 (2)	0.62115 (17)	0.6703 (4)	0.0259 (5)	
H13A	0.6153	0.6529	0.6478	0.039*	
H13B	0.5651	0.5611	0.6924	0.039*	
H13C	0.5108	0.6446	0.7803	0.039*	
C14	−0.0733 (3)	0.31310 (18)	1.2189 (4)	0.0340 (7)	
H14A	−0.1489	0.3273	1.2525	0.051*	
H14B	−0.0248	0.3222	1.3275	0.051*	
H14C	−0.0696	0.2533	1.1802	0.051*	
O1	0.47461 (14)	0.62845 (11)	0.5063 (2)	0.0238 (4)	
O2	−0.03855 (15)	0.36749 (13)	1.0649 (3)	0.0334 (4)	

### Atomic displacement parameters (Å^2^)

**Table:**

	*U*^11^	*U*^22^	*U*^33^	*U*^12^	*U*^13^	*U*^23^
I1	0.02789 (11)	0.02800 (11)	0.02336 (11)	0.00115 (6)	0.00705 (7)	0.00536 (6)
C1	0.0225 (12)	0.0197 (11)	0.0200 (12)	−0.0003 (9)	0.0043 (9)	0.0001 (9)
C2	0.0185 (11)	0.0174 (11)	0.0205 (11)	−0.0006 (9)	0.0036 (9)	−0.0022 (9)
C3	0.0133 (10)	0.0184 (11)	0.0156 (11)	0.0032 (8)	−0.0027 (8)	−0.0051 (8)
C4	0.0206 (11)	0.0204 (11)	0.0200 (12)	−0.0002 (9)	0.0016 (9)	−0.0006 (9)
C5	0.0201 (12)	0.0284 (13)	0.0267 (13)	−0.0055 (10)	−0.0031 (10)	0.0035 (10)
C6	0.0220 (12)	0.0302 (13)	0.0226 (12)	−0.0008 (10)	−0.0047 (10)	0.0019 (10)
C7	0.0224 (11)	0.0176 (11)	0.0209 (12)	−0.0035 (9)	−0.0037 (9)	−0.0022 (9)
C8	0.0206 (12)	0.0229 (12)	0.0222 (12)	−0.0008 (9)	−0.0026 (9)	−0.0011 (10)
C9	0.0248 (13)	0.0243 (12)	0.0208 (12)	0.0030 (10)	−0.0053 (10)	−0.0015 (10)
C10	0.0290 (13)	0.0228 (12)	0.0188 (12)	−0.0031 (10)	−0.0017 (10)	0.0026 (10)
C11	0.0213 (12)	0.0226 (12)	0.0239 (12)	−0.0018 (9)	0.0008 (10)	0.0013 (10)
C12	0.0211 (12)	0.0197 (11)	0.0259 (13)	0.0017 (9)	−0.0010 (9)	0.0030 (10)
C13	0.0215 (12)	0.0306 (13)	0.0257 (13)	−0.0047 (10)	0.0006 (10)	−0.0071 (11)
C14	0.0318 (15)	0.0372 (16)	0.0331 (16)	−0.0057 (11)	0.0109 (12)	0.0092 (12)
O1	0.0195 (8)	0.0284 (9)	0.0236 (9)	−0.0066 (7)	0.0013 (7)	−0.0004 (7)
O2	0.0239 (9)	0.0370 (11)	0.0396 (11)	0.0024 (8)	0.0080 (8)	0.0157 (9)

### Geometric parameters (Å, °)

**Table:**

I1—C1	2.098 (2)	C9—C10	1.393 (4)
C1—C6	1.382 (3)	C9—H9A	0.9600
C1—C2	1.400 (3)	C10—C11	1.388 (4)
C2—C3	1.363 (3)	C10—H10A	0.9600
C2—O1	1.364 (3)	C11—O2	1.370 (3)
C3—C4	1.416 (3)	C11—C12	1.399 (3)
C3—H3A	0.9600	C12—H12A	0.9600
C4—C5	1.397 (3)	C13—O1	1.436 (3)
C4—C7	1.490 (3)	C13—H13A	0.9599
C5—C6	1.391 (4)	C13—H13B	0.9599
C5—H5A	0.9600	C13—H13C	0.9599
C6—H6A	0.9600	C14—O2	1.429 (3)
C7—C12	1.386 (3)	C14—H14A	0.9599
C7—C8	1.402 (3)	C14—H14B	0.9599
C8—C9	1.382 (4)	C14—H14C	0.9599
C8—H8A	0.9600		
			
I1···O1^i^	3.408 (2)		
			
C6—C1—C2	121.0 (2)	C8—C9—H9A	119.1
C6—C1—I1	119.76 (18)	C10—C9—H9A	119.1
C2—C1—I1	119.25 (17)	C11—C10—C9	118.5 (2)
C3—C2—O1	124.5 (2)	C11—C10—H10A	120.7
C3—C2—C1	119.0 (2)	C9—C10—H10A	120.7
O1—C2—C1	116.5 (2)	O2—C11—C10	124.8 (2)
C2—C3—C4	121.6 (2)	O2—C11—C12	115.0 (2)
C2—C3—H3A	119.2	C10—C11—C12	120.1 (2)
C4—C3—H3A	119.2	C7—C12—C11	120.8 (2)
C5—C4—C3	118.3 (2)	C7—C12—H12A	119.6
C5—C4—C7	121.0 (2)	C11—C12—H12A	119.6
C3—C4—C7	120.7 (2)	O1—C13—H13A	109.5
C6—C5—C4	120.4 (2)	O1—C13—H13B	109.5
C6—C5—H5A	119.8	H13A—C13—H13B	109.5
C4—C5—H5A	119.8	O1—C13—H13C	109.5
C1—C6—C5	119.7 (2)	H13A—C13—H13C	109.5
C1—C6—H6A	120.1	H13B—C13—H13C	109.5
C5—C6—H6A	120.1	O2—C14—H14A	109.5
C12—C7—C8	119.1 (2)	O2—C14—H14B	109.5
C12—C7—C4	120.4 (2)	H14A—C14—H14B	109.5
C8—C7—C4	120.4 (2)	O2—C14—H14C	109.5
C9—C8—C7	119.4 (2)	H14A—C14—H14C	109.5
C9—C8—H8A	120.3	H14B—C14—H14C	109.5
C7—C8—H8A	120.3	C2—O1—C13	117.71 (19)
C8—C9—C10	121.9 (2)	C11—O2—C14	117.4 (2)
			
C6—C1—C2—C3	−1.6 (4)	C3—C4—C7—C8	−43.5 (3)
I1—C1—C2—C3	178.93 (16)	C12—C7—C8—C9	0.6 (4)
C6—C1—C2—O1	177.8 (2)	C4—C7—C8—C9	−178.3 (2)
I1—C1—C2—O1	−1.7 (3)	C7—C8—C9—C10	−0.8 (4)
O1—C2—C3—C4	−178.0 (2)	C8—C9—C10—C11	0.0 (4)
C1—C2—C3—C4	1.3 (3)	C9—C10—C11—O2	−178.8 (2)
C2—C3—C4—C5	−0.4 (3)	C9—C10—C11—C12	1.0 (4)
C2—C3—C4—C7	178.5 (2)	C8—C7—C12—C11	0.4 (4)
C3—C4—C5—C6	−0.4 (4)	C4—C7—C12—C11	179.2 (2)
C7—C4—C5—C6	−179.2 (2)	O2—C11—C12—C7	178.7 (2)
C2—C1—C6—C5	0.9 (4)	C10—C11—C12—C7	−1.2 (4)
I1—C1—C6—C5	−179.64 (19)	C3—C2—O1—C13	−3.3 (3)
C4—C5—C6—C1	0.1 (4)	C1—C2—O1—C13	177.3 (2)
C5—C4—C7—C12	−43.6 (3)	C10—C11—O2—C14	2.9 (4)
C3—C4—C7—C12	137.6 (2)	C12—C11—O2—C14	−176.9 (2)
C5—C4—C7—C8	135.3 (3)		

Symmetry codes:  (i) *x*, −*y*+3/2, *z*−1/2.
